# Low Bacterial Diversity and Nitrate Levels in Cores from Deep Boreholes in Pristine Karst

**DOI:** 10.3390/life14060677

**Published:** 2024-05-24

**Authors:** Janez Mulec, Sara Skok, Lejla Pašić

**Affiliations:** 1 Karst Research Institute, Research Centre of the Slovenian Academy of Sciences and Arts, Titov trg 2, 6230 Postojna, Slovenia; sara.skok@zrc-sazu.si; 2 UNESCO Chair on Karst Education, University of Nova Gorica, 5271 Vipava, Slovenia; 3 Sarajevo Medical School, University Sarajevo School of Science and Technology, Hrasnička cesta 3a, 71000 Sarajevo, Bosnia and Herzegovina; lejla.pasic@ssst.edu.ba

**Keywords:** karst, subsurface, sediments, nitrogen

## Abstract

This study investigates the nitrate gradients within the deep biosphere of karst carbonate rocks and their resident microbiota. Samples were taken from borehole cores at depths down to 350 m below the surface, collected during geological site investigations for proposed railway tunnels and analysed using 16S rRNA amplicon sequencing. 16S rRNA amplicon sequencing analysis revealed relatively low microbial diversity, which can serve as a reliable indicator of the pristine nature of deep karst. However, some local hotspots of diversity are independent of depth. Pseudomonadota dominated the samples, with Gammaproteobacteria dominating at the class level. The low nitrate content in deep karst, in contrast to higher values closer to the surface, serves as an additional marker of its undisturbed and unpolluted status. Based on the prediction of functional profiles from 16S rRNA sequencing data, nitrates remain low due to indigenous microbial denitrification and assimilatory nitrate reduction. Pathways related to nitrogen fixation, ammonia assimilation, and nitrification were not confirmed. When elevated nitrate levels are observed in karst, they are most probably related to anthropogenic activities. Environmental factors other than depth and nitrate content play an important role in shaping bacterial communities.

## 1. Introduction

Microbiota in the deep biosphere has received increasing attention as an important participant in geochemical cycling [1]. With increasing depth, it exceeds colonisation of the critical zone, which extends in the upper layers of the Earth’s crust, from the vegetation canopy, through the soils or pedosphere, into the unsaturated and saturated bedrock including aquifers [2]. The study of microbial communities in the subsurface is challenging because of the inaccessibility of their habitats. Nevertheless, natural caves that enable human access into the subsurface provide non-destructive windows to gain insights into their environment.

To obtain valuable information from the deep biosphere, drilling of boreholes and subsequent analysis of borehole cores is commonly used [3]. Cored boreholes, whether drilled vertically, inclined, or horizontally, provide shafts into the ground that can be used for a variety of purposes, including rock testing as part of geotechnical investigations [4]. Using this approach, microbiologists can gain access to the subsurface microbiota and gather essential data to support investigation projects related to potential underground constructions, such as proposed nuclear waste disposal sites [5].

Some geological settings can offer environmental conditions favourable for microbial colonisation, primarily due to the presence of sufficient water and nutrients. An outstanding example of such settings is provided by soluble carbonate rocks such as limestone and dolomite. These rocks are susceptible to the effects of aqueous dissolution, leading to the development of karst features, including accessible caves and a variety of smaller voids that contribute to underground drainage systems. For example, the integrity of karst rocks is commonly disrupted by fractures, which increase the rock permeability and, in some cases, widen into caves. Sediments derived from weathered rocks, loessic clay, and organic material tend to accumulate within these voids, commonly creating fill deposits. Such diverse materials originate from the surface or can be generated in situ by way of abiotic or biotic weathering processes [6,7]. Vertical and inclined fractures enlarged by karstification act as conduits allowing transmission of dissolved and particulate material between the surface and the subsurface [8]. This connection plays a key role in facilitating the transport of contaminants from higher to lower strata. Such dissolution channels can provide relatively open routes for the transmission of potential pollutants to areas deep underground, where ultimately they can reach karst springs and affect water supply quality [9]. An example of this type of activity is observed with nitrates, which are highly soluble and readily leached from the soil into underlying strata and eventually transferred into the groundwater [10]. Nitrogen plays a crucial role in karst areas and in the deep biosphere, influencing microbial communities and ecosystem processes. Nitrogen enters karst areas mainly through agricultural runoff and atmospheric deposition, often in the form of nitrate. This infiltration has significant ecological impacts, including changes in the composition and function of microbial communities in subsurface ecosystems. Elevated nitrate levels in sediment from a karst cave have been shown to affect the diversity and metabolic pathways of microbial communities, e.g., nitrate reducers and ammonium oxidisers [11]. High concentrations of nitrates pose significant risks to human health [12].

This study set out to assess the nitrate gradient within carbonate rocks in the deep biosphere and investigate its correlation with the associated microbiota. The investigation aimed (1) to explore the microbial diversity and its metabolic potential on nitrogen cycling due to the increasing issue of elevated nitrates in karst areas and (2) to provide an overview of microbial colonisation within the deep karst biosphere. Samples used in the study were obtained from cores extracted from cored boreholes, extending to depths of 350 m, during the geological investigations conducted prior to the construction of railway tunnels within the karst region in Slovenia.

## 2. Materials and Methods

### 2.1. Site Description and Sampling

The government of Slovenia has undertaken a project to address the growing demand for goods transportation by constructing a new 27 km railway route connecting the port of Koper (0 m a.s.l.) to the mainland town of Divača (435 m a.s.l.). Beyond Divača, the trains will continue to utilise the existing railway network. The project is expected to be completed by the end of 2025. Due to the specific landscape characteristics of the region, the new railway route will encompass a total of eight tunnels, covering a combined length of 20.5 km, between the port of Koper and Divača. Notably, two of these tunnels, named T1 and T2, will be traversing through karst rock successions. The railway route near Divača, initially on top of the Classical Karst plateau, will traverse about 3 km at the surface before entering the first tunnel, T1, which has a length of 6.7 km. This tunnel will terminate in the upper part of the Glinščica valley. Subsequently, the railway will cross a bridge and then enter the second tunnel, T2, which is also located in a karst region and has a length of 6 km. At certain points along the route, the railway will run at a depth of more than 300 m beneath the land surface. This ambitious railway infrastructure project is intended to facilitate smoother connectivity between the port of Koper and the mainland town of Divača while navigating through the challenging terrain of the karst region [13].

**Figure 1:**
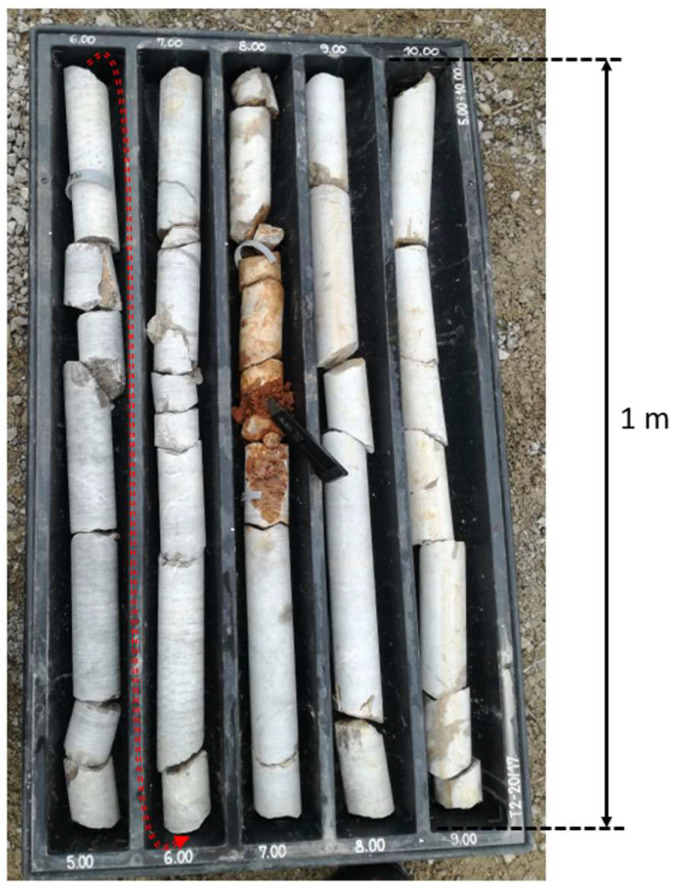
An example of limestone cores from −5 to −10 m (T2_20), each with a single length of 1 m, and sampling of red clay in the fracture (Table S1 in Supplementary Materials). The superimposed red arrow indicates the core relationships (orientation and continuity) in the vertical profile.

Before commencing the actual construction of the tunnels for the new railway route, an extensive geological and karstological study of the terrain was undertaken. The main objectives of this investigation were to identify geological structures, understand their spatial characteristics, and locate any previously unknown underground voids (including caves) in the area. During the planning phase, significant efforts were made to ensure the avoidance and preservation of any large, known karst caves within the preferred development corridor. In the final phases of the investigation, the methodology involved borehole drilling and analysis of the retrieved cores [14]. These cores revealed a compact rock succession interspersed with distinct zones of softer fill, interrupted by fissures at varied spacings (Figure 1). The vertical profile of the cores, reaching –350 m in depth, revealed the presence of soft material at different levels within the boreholes. Aseptic sampling of the soft material was carried out in the field as soon as the core was accessible after drilling to avoid drying out and to preserve the natural moisture content and consistency of the samples. The volume of voids filled with the material was not estimated in situ to avoid possible contamination. To prevent potential contamination resulting from aspects of the drilling process, including the use of cooling water, the outermost layer of the soft material from the core was discarded. An approximately two-millimetre-thin layer of the material was cut off with a sterile knife and only the inner part was used for the analyses. Because the quantity of fillings at some depths was limited, only a small amount of material (a minimum of 0.018 g) could be collected from each sample. Samples were collected from the cores of four boreholes, two (designated 12, 13) at the location of tunnel T1 (T1_12: 45°38′53.3″ N 13°56′01.9″ E, depth: 250 m, T1_13: 45°38′10.4″ N 13°55′29.4″ E, depth: 350 m) and two (designated 19, 20) at the location of tunnel T2 (T2_19: 45°33′43.2″ N 13°52′44.8″ E, depth: 250 m, T2_20: 45°33′34.6″ N 13°52′38.0″ E, depth: 150 m). The sampling took place during several drilling campaigns between February and April 2018.

### 2.2. Nitrates Measurement and DNA Isolation

The first laboratory analysis performed on all 66 samples involved estimating the levels of nitrates. If sufficient material remained after the nitrate estimation, DNA isolation was also carried out on the same samples. Additionally, five samples of different soils from sites on the karst surface were collected for comparison with the nitrate levels estimated within the cores. These soil sample localities included Dutovlje (meadow close to vineyard: 45°45′38.8″ N 13°49′5.0″ E), Lokev (cabbage garden, spinach garden, and garden after application of fertiliser: 45°39′37.0″ N 13°56′15.5″ E), and Šepulje (meadow: 45°45′14.5″ N 13°52′36.6″ E).

In consideration of the limited quantity of sample material available from the borehole cores, a field determination kit (LAQUAtwin NOD-B-742, Horiba, Tokyo, Japan) was employed to estimate nitrate concentrations (0.3 mL is the minimum sample volume). To elute nitrates from the samples, two parts (*w*/*v*) of distilled water were added to the tubes containing the samples, which were then vortexed vigorously. Afterwards, the samples were centrifuged at 13,000 RPM for 2 min to remove any suspended clay material and minimise the possibility of its interference with the nitrate measurement. The nitrate concentration measurement was conducted according to the field determination kit instruction manual. Two measurements were performed for each sample, and the average value was calculated and recorded. The nitrate concentration was expressed in parts per million (ppm). To validate the accuracy of the results obtained using the field determination kit (specified by the producer for low NO_3_-N range from 1.4 to 2200 ppm, accuracy ±10% of reading value), a statistically significant correlation (*p* < 0.05) was established between the values obtained from the LAQUAtwin NOD-B-742, and example results were obtained via a spectrometric method using sulfosalicylic acid (ISO 7890-3: 1988) [15]. Finally, the nitrate concentrations recorded were expressed in mg per kg of fill material and plotted against the depths of the samples within the boreholes to create a comprehensive profile, allowing for a clearer illustration and understanding of the distribution of nitrates throughout the vertical extent of the cores.

DNA isolation was performed using the DNeasy PowerSoil^®^Pro kit (Qiagen GmbH, Hilden, Germany) following the manufacturer’s instructions. The PowerSoilPro DNA isolation kit (Qiagen) was employed to isolate DNA from the fillings. The rationale behind selecting this kit was its ability, as stated by the manufacturer, to address challenges associated with DNA adsorption on clay particles effectively and to remove inhibitors commonly found in soil, such as humic substances. Nevertheless, limitations were encountered related to the quantity of available fill material from some of the core samples, and this restricted the kit’s ability to obtain sufficient high-quality DNA for the comprehensive sequencing of all samples. Thus, 14 out of 66 samples were sequenced. The extracted DNA was subsequently assessed for purity and concentration using spectrophotometric analysis. This analysis involved measuring the absorbances at 260 nm and 280 nm on a Nanodrop ND-2000 spectrophotometer (Peqlab, Erlangen, Germany) (Table S1 in Supplementary Materials).

Illumina MiSeq sequencing, 16S rRNA amplicon metagenomics data processing, and the identification of functional metabolic pathways were undertaken. The preparation of libraries, sequencing, and part of the data analysis were conducted by Microsynth AG (Balgach, Switzerland), as described below.

### 2.3. Library Preparation and Sequencing

The V3 and V4 regions of the bacterial 16S rRNA gene were sequenced using two-step Nextera PCR libraries with the primer pair 341F (5′-CCT ACG GGN GGC WGC AG-3′) and 802R (5′-GAC TAC HVG GGT ATC TAA TCC-3′). The Illumina MiSeq platform with a v2 500 cycles kit was utilised for the sequencing of the PCR libraries.

#### 2.3.1. Data Preprocessing

The paired-end reads that passed Illumina’s chastity filter were subjected to de-multiplexing and trimming of Illumina adaptor residuals using the MiSeq reporter software v2.6. The quality of the reads was assessed using FastQC version 0.11.8 [16]. Locus-specific V34 primers were trimmed from the sequencing reads using cutadapt v2.3 [17].

#### 2.3.2. Merging and Noise Reduction

Trimmed forward and reverse reads of each paired-end read were merged to reconstruct the sequenced molecule in silico, considering a minimum overlap of 15 bases using the software USEARCH version 11.0.667 [18]. Merged sequences were quality-filtered, allowing a maximum of one expected error per merged read. Reads containing ambiguous bases or outliers in the amplicon size distribution were discarded. Noise reduction was performed using the UNOISE algorithm [19] implemented in USEARCH [18], generating operational taxonomic units (OTUs) and removing singletons and chimaeras.

#### 2.3.3. Taxonomic Classification

OTUs were generated at 97% sequence similarity for the genus level and at 99% similarity for the species level. The resulting OTU abundance table was filtered for potential bleed-in contaminations using the UNCROSS algorithm [20], and abundances were adjusted for 16S rRNA copy numbers using the UNBIAS algorithm [21]. Taxonomies were predicted based on comparison against the reference sequences of the RDP 16S rRNA database [22], using the SINTAX algorithm [23] implemented in USEARCH with a minimum confidence threshold of 0.5.

#### 2.3.4. Diversity Analysis

Alpha diversity was estimated using the Shannon index, while beta diversity was calculated using the weighted Unifrac distance method on the basis of rarefied OTU abundance counts per sample. Detrended correspondence analysis (DCA) was used to explore patterns of inter-sample relationships. Alpha and beta diversity calculations and the rarefaction analysis were performed with the R software packages phyloseq v1.26.1 [24] and vegan v2.5-5.

#### 2.3.5. Rarefaction and Functional Profiling

Rarefaction curves were determined based on the Shannon index. Functional profiles were predicted using the picrust2 v2.1.4-b software [25] and its integrated MetaCyc database, which suggests metabolic pathways present in an organism based on the inferred catalytic activities of encoded proteins [26].

#### 2.3.6. Data Visualisation

The vegan community ecology package [27] and Bioconductor repository [28] were used within the R software to produce heatmaps.

#### 2.3.7. Data Availability

The data presented in this study are openly available in the NCBI Sequence Read Archive under the BioProject accession number PRJNA1008434 corresponding to BioSample numbers SAMN37119913-SAMN37119926.

## 3. Results and Discussion

### 3.1. Microbiota in Cores

An investigation into the microbial communities present in fillings derived from four core samples extracted at varying depths was conducted utilising 16S rRNA amplicon sequencing analysis. After applying quality filtration to the sequence readings, a total of 670,813 reads were obtained from the fourteen samples studied (Table 1). The subsequent analysis of operational taxonomic units (OTUs) revealed different microbial diversity within the samples. The number of OTUs varied from 27 in sample T1_13 at a depth of 314 m to 234 in sample T1_13 at a depth of 292 m. The analysis revealed no clear general trend, such as an increase in OTUs or diversity with increasing depth. Additionally, rarefaction curves indicated that the diversity within the samples had been sampled sufficiently (Figure S1 in Supplementary Materials). This finding suggests that the sequencing effort was effective in capturing a representative portion of the bacterial diversity present in the studied fill materials at different depths.

**Figure 2:**
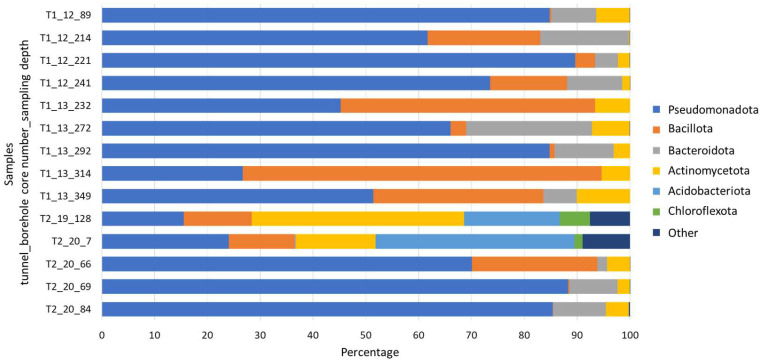
Relative abundance of bacterial phyla in the studied samples. Phyla that represented <5% of the microbial communities in the samples were classified as “Other”.

Members of 15 bacterial phyla were identified within the microbial communities of the fill materials (Figure 2). Predominantly, the bacteria in most samples belonged to the phylum Pseudomonadota (abundance range: 45.2–89.6%). However, specific exceptions were observed; for instance, the filling samples T1_13, obtained at depths of 232 and 314 m, were dominated by members belonging to the phylum Bacillota (48.2% and 67.9%, respectively). Another exception was found in the sample T2_20, collected at a depth of 7 m, wherein microorganisms affiliated with the phylum Acidobacteriota prevailed (37.6%). Furthermore, sample T2_19 from a depth of 128 m exhibited a dominance of bacteria from the phylum Actinomycetota (40.2%). Phyla representing less than 5% of the samples and being present in no more than two samples were categorised as “Other”, and these encompassed Armatimonadota, Chlamydiota, Euryarchaeota, Gemmatimonadota, Latescibacteria, Nitrospirota, Parcubacteria, Planctomycetota, and Verrucomicrobiota.

**Figure 3:**
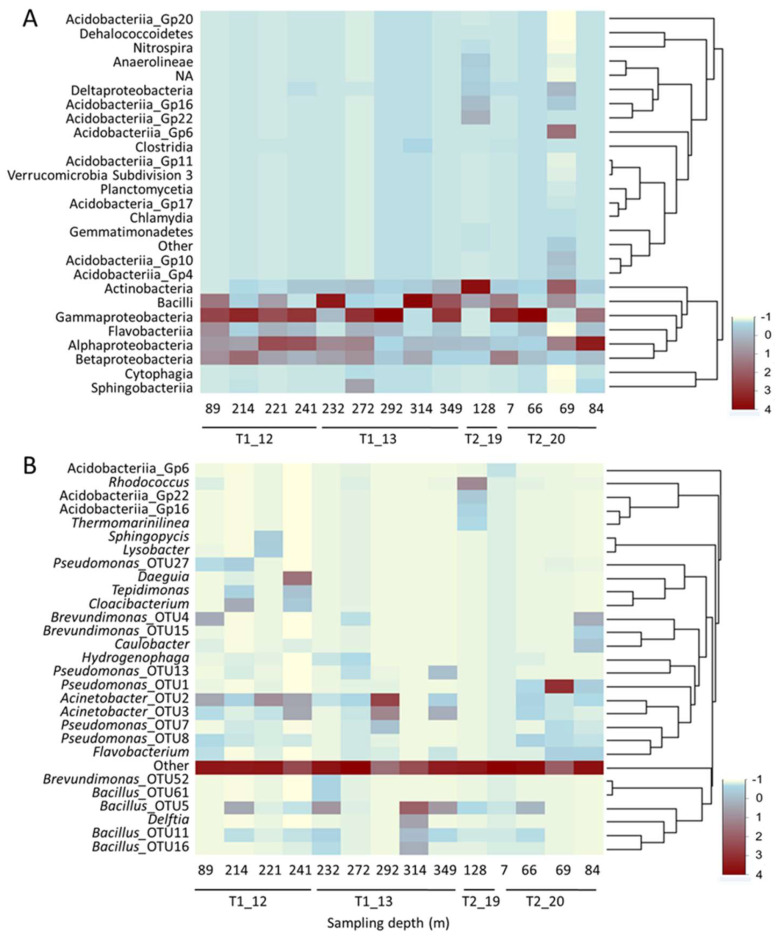
Heatmap analysis of relative abundance of members of microbial communities in fillings of borehole core samples at class (**A**) and genus (**B**) levels. Phylotypes whose relative abundance was >1% in at least one of the samples are listed.

Among the 39 classes detected in this study, 27 exhibited abundances surpassing one percent in at least one of the samples (Figure 3A). Ten samples were dominated by various proportions of Pseudomonadota classes. Within the Pseudomonadota phylum, nine samples were dominated by Gammaproteobacteria (abundance range: 29.8–75.6%), while sample T2_20, collected at a depth of 84 m, was dominated by Alphaproteobacteria (49.5%). Other abundant classes encompassed Bacilli, a representative class of Bacillota, which prevailed in samples T1_13 obtained at depths of 232 m (48.2%) and 314 m (65.7%), respectively. Additionally, Actinobacteria dominated sample T2_19 acquired at a depth of 128 m (40.2%). The dominance of Acidobacteria in sample T2_20 collected at a depth of 69 m resulted from the abundance of six distinct acidobacterial classes.

Altogether, 180 genera were identified in all the samples. At the genus level (Figure 3B), the dominance of a single phylotype was observed only rarely among the samples. However, an exception was found in sample T2_20, obtained at a depth of 69 m, which was dominated by *Pseudomonas*_OTU1 (44.3%), corresponding to *P. brenneri*, a species previously isolated from natural mineral waters and belonging to the *Pseudomonas fluorescens* group [29]. Other remarkably abundant phylotypes included *Acinetobacter*_OTU2 (36.4%), corresponding to *A. harbinensis*, known for its nitrifying ability [30], which dominated sample T1_13 taken at a depth of 292 m. Additionally, *Bacillus*_OTU5 (28.6%), corresponding to *B. pseudofirmus*, previously isolated from a hyperalkaline spring [31], dominated sample T1_13 obtained at a depth of 314 m. Several other OTUs were notably abundant, albeit to a lesser extent, and included *Daeguia* (19.9%), which dominated sample T1_12, obtained at a depth of 241 m, and corresponded to *Daeguia caeni* [32], isolated from sludge of a textile dye works, and *Rhodococcus* T2_19, obtained at a depth of 128 m.

**Figure 4:**
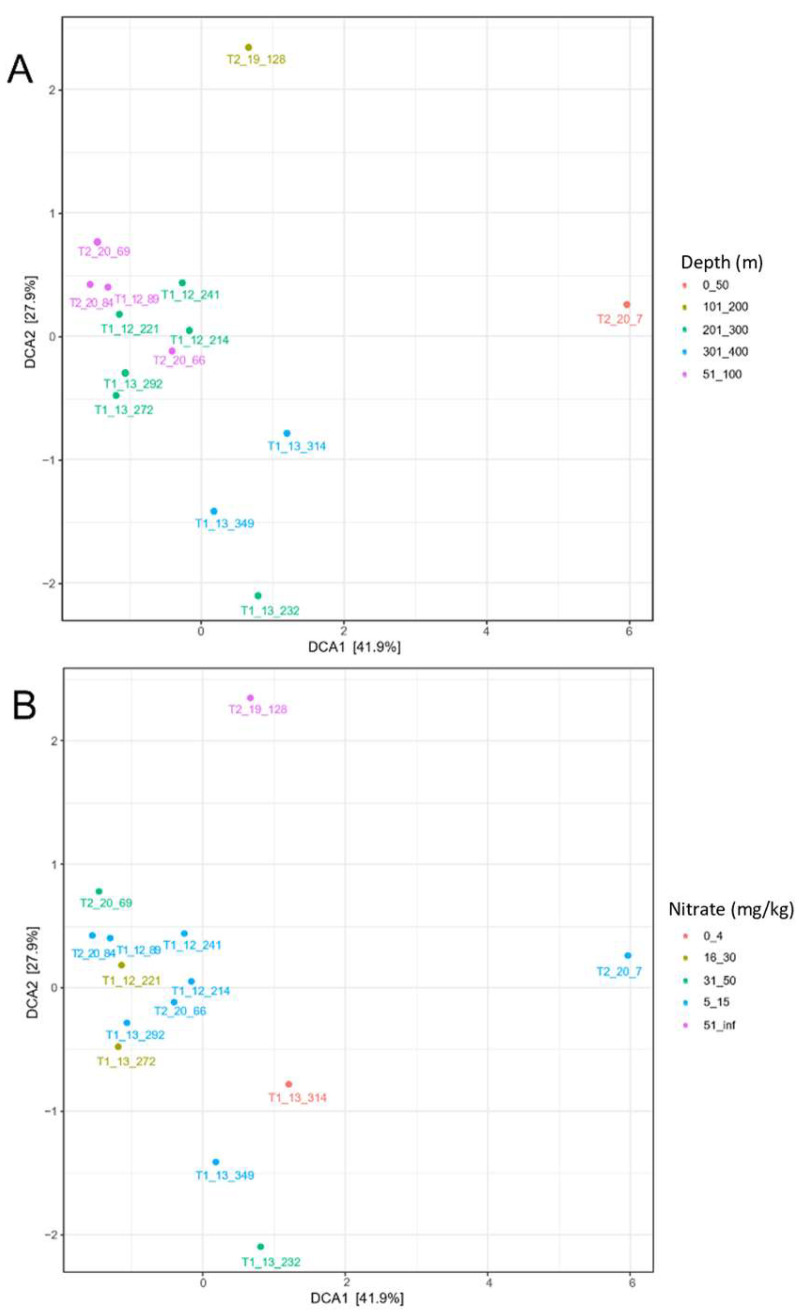
The detrended correspondence analysis of weighted Unifrac distances based on sampling depth (**A**) and nitrate concentration (**B**). Sample—tunnel borehole core number_sampling depth (m).

In the subsequent analysis, the samples were categorised into five groups according to their respective sampling depths: samples collected at depths from 0 to 50 m, from 51 to 100 m, from 101 to 200 m, from 201 to 300 m, and from 301 to 400 m (Figure 4A). The detrended correspondence analysis of weighted Unifrac distances revealed similarities among the bacterial communities of certain samples based on the sampling depth gradient (e.g., 51–100 m and 201–300 m). However, this trend was not observed consistently across all samples. Next, the samples were grouped into five categories based on the nitrate concentration in the fill material, namely 0–4 mg/kg, 5–15 mg/kg, 16–30 mg/kg, 31–50 mg/kg, and >51 mg/kg (Figure 4B). No distinct grouping pattern based on nitrate gradient of the samples was observed. Thus, it was concluded that the differences in beta diversity among the samples could not be explained adequately either by the sampling depth or by the nitrate gradient.

### 3.2. Nitrates and Functional Metabolic Profiles

**Figure 5:**
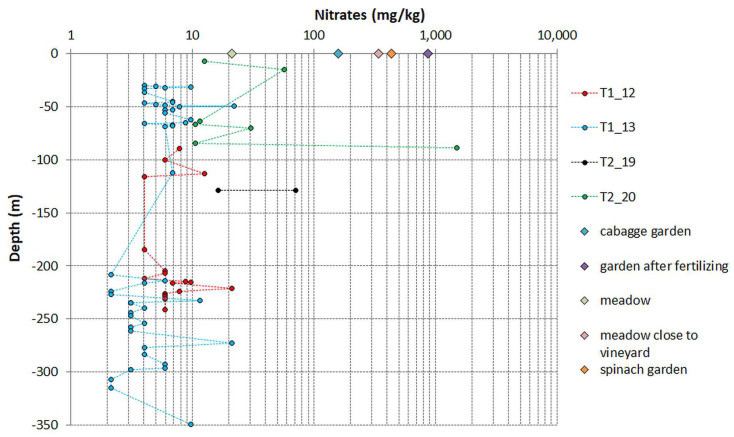
Nitrate concentration in fillings from four cores (tunnel_core) in a vertical profile compared to soils collected at the surface of the karst.

Nitrates were assessed in the samples to investigate the presence of a concentration gradient or localised areas with elevated concentrations, potentially caused by water infiltration, given their susceptibility to leaching from the soil into lower layers. The concentrations of nitrates from borehole core fills were found to be relatively low compared to those found in surface samples, especially those obtained from human-impacted soils, such as in gardens and meadowland close to vineyards (ranging from 158 to 870 mg/kg). In the core profiles, a consistent pattern of nitrate concentration was observed for samples from cores T2_12 (ranging from 4 to 21 mg/kg, with an average of 7 mg/kg ± 4 mg/kg) and T2_13 (ranging from 2 to 22 mg/kg, with an average of 6 mg/kg ± 4 mg/kg). However, this uniformity was less pronounced for samples from T1_19 (ranging from 16 to 71 mg/kg, with an average of 44 mg/kg ± 39 mg/kg) and T1_20 (ranging from 11 to 1504 mg/kg, with an average of 234 mg/kg ± 560 mg/kg). Notably, the highest nitrate concentration of 1504 mg/kg was detected at a depth of 89 m in sample T2_20 (Figure 5). This finding highlights the presence of localised areas with significantly elevated nitrate concentrations, possibly attributed to peculiarities of filling material or specific environmental conditions attributed to microbial metabolism.

Nitrates are part of the nitrogen cycle, which is linked to microbial metabolism. To analyse the functional metabolic pathways of microbes using the 16S rRNA amplicon sequencing data, MetaCyc pathway abundances were predicted, resulting in a total of 404 pathways. Within this dataset, specific attention was given to metabolic processes related to nitrogen cycling, including nitrogen fixation, assimilation, ammonification, nitrification, denitrification, dissimilatory reduction of nitrate to ammonia, and anaerobic ammonia oxidation.

The analysis revealed the functional genetic potential presence of bacteria engaged in denitrification (nitrate reduction I, BioCyc ID: DENITRIFICATION-PWY) in all samples. This pathway occurs under oxygen-depleted conditions in various groups of bacteria, archaea, and fungi [33,34] and finally forms nitrogen gas. The widespread occurrence of denitrification pathways in the samples suggests the prevalence of oxygen-depleted conditions in the fill materials. Such conditions arise due to limited oxygen diffusion, microbial respiration, and ultimately the presence of organic matter that can facilitate the development of anoxic zones.

Additionally, predicted functional pathways indicated the existence of microorganisms possessing enzymes involved in assimilatory nitrate reduction (nitrate reduction VI, BioCyc ID: PWY490-3) across the samples. This pathway is present in cyanobacteria, typified by the enzymes from *Synechococcus* sp. PCC7942 [35]. The presence of cyanobacteria in the deep subsurface environment is intriguing and carries important implications. Cyanobacteria are known as phototrophic organisms, relying on light as an energy source for photosynthesis. If we exclude potential sample contamination, their presence in such subsurface environments suggests that they possess a remarkable ability to adapt and survive under suboptimal living conditions. Additionally, their ability to engage in nitrogen assimilation in the deep subsurface underscores the potential significance of these microorganisms in contributing to nitrogen cycling processes in this unique habitat. Their presence in the deep subsurface could also indicate a good connection to the surface.

The presence of the specific metabolic pathway of aerobic denitrification (BioCyc ID: PWY-7084) in sample T2_20, obtained at a depth of 7 m, is noteworthy. This pathway is commonly observed in autotrophic ammonia oxidisers [36], as well as in many methane-oxidizing bacteria [37]. Notably, this pathway leads to the formation of nitrogen in fully oxic to hypoxic conditions. The presence of aerobic denitrification in this sample suggests that the environment might experience fluctuating oxygen concentrations and have access to reduced carbon sources [38]. This interpretation appears plausible because the site is relatively close to the surface, possibly providing a higher potential for the inclusion of available organic carbon compared to other sites. However, no functional genetic potential for metabolic pathways related to nitrogen fixation, ammonia assimilation, or nitrification was identified in any of the samples (Table 2). There are no comparable studies on the microbiota from borehole cores, but the results from caves show an influence of nitrate on microbial communities and the importance of nitrogen cycling for the functioning of the karst ecosystem together with methane metabolism and carbon fixation [11].

The results showed that in the case of pristine deep karst environments, the bacterial diversity is low in the deep karst biosphere, but local diversity hotspots exist irrespective of depth. Due to their abundance and metabolic versatility, Pseudomonadota are probably one of the key players in this environment, but even the role of Cyanobacteriota in the overall ecosystem dynamic should not be underestimated. In addition to depth, other factors are also important for shaping the community structure, and nitrates are not among the critical ones. The concept of autochthonous microbial endokarst communities (AMECs) consisting of specific bacterial species within groups of Acidobacteria, Nitrospira, and Deltaproteobacteria, as observed in karst springs [39], should undergo further investigation in the case of the microbiota in fractures within deep karst rocks. Longer water residence time favours the development of AMECs [40,41], but this might only apply to microbial habitats connected by water flow, and in the case of locally isolated habitats, community structure is likely to change over time. However, the idea of a “core microbiome” in deep karst should be explored further. Nitrate levels are low in deep karst situations compared to the near-surface values. This situation can be attributed to microbial denitrification and assimilatory nitrate reduction and to the absence or low level of microbial nitrification. Nitrification of organic nitrogen contributes to increased nitrate levels in karst impacted by anthropogenic activities, e.g., contamination from urban wastewater [42]. The indigenous microbiota probably does not contribute to the elevation of nitrate levels, and thus, they should generally be low. Additionally, low diversity reflects low metabolic potential in this environment.

## 4. Conclusions

An overall low background level of microbial diversity, as evidenced in the cores of deep boreholes, can be considered to characterise the average conditions expected in a pristine deep karst environment. However, there are local hotspots of diversity within the karst regardless of depth. Because of their abundance, Pseudomonadota are thought to play a key role in this ecosystem, but this should be confirmed further by analysing their metabolic activity. Another parameter indicating the pristine nature of deep karst and the absence of anthropogenic impacts is the low nitrate content compared to levels at the land surface. Nitrogen is generally a limiting nutrient for biota [43]. It seems that microbiota in deep karst can preserve some nitrogen from nitrates by assimilatory nitrate reduction despite denitrification, which implies nitrogen loss in the forms of nitrous oxide (N_2_O) and dinitrogen (N_2_). Such a strategy is even more important in environments such as karst because, as a landscape with little or no soil cover, karst can provide only limited organic inputs to the lower layers. The analysis of the content and properties of fills in cores from boreholes drilled in karst environments is crucial for understanding the current state and future trends because the fills serve as repositories for organic and inorganic substances, as well as for microorganisms.

## Figures and Tables

**Table 1:** Number of reads, rarefied depth, number of OTUs, and Shannon index values for the studied samples.

Sample tunnel_core_depth (m)	No. Reads	No. OTUs	Shannon Index
T1_12_89	33,155	219	3.67
T1_12_214	7712 ^1^	51	3.29
T1_12_221	27,049	99	3.57
T1_12_241	13,268	38	2.88
T1_13_232	27,048	39	2.98
T1_13_272	100,857	196	4.36
T1_13_292	78,523	234	2.61
T1_13_314	17,360	27	2.34
T1_13_349	8386	37	2.95
T2_19_128	43,342	69	3.24
T2_20_7	73,559	193	4.9
T2_20_66	19,641	102	3.58
T2_20_69	104,557	196	2.83
T2_20_84	116,356	230	3.77

^1^ Value is equal to rarified depth.

**Table 2:** Abundances of metabolic pathways (copies per million reads) involved in nitrogen cycling.

Sample tunnel_core_depth (m)	Denitrification	Assimilatory Nitrate Reduction	Aerobic Denitrification
T1_12_89	3460	2880	0
T1_12_214	1570	212	0
T1_12_221	1480	637	0
T1_12_241	2480	461	0
T1_13_232	2330	689	0
T1_13_272	21,900	27,400	0
T1_13_292	4490	9580	0
T1_13_314	0 ^1^	369	0
T1_13_349	991	560	0
T2_19_128	906	307	0
T2_20_7	913	3870	820
T2_20_66	2280	420	0
T2_20_69	104,557	196	2.83
T2_20_84	116,356	230	3.77

^1^ 0.315.

## Data Availability

The original data presented in the study are openly available in Sequence Read Archive under the BioProject accession number PRJNA1008434 corresponding to BioSample numbers SAMN37119913-SAMN37119926.

## Supplementary Materials

The following supporting information can be downloaded at: https://www.mdpi.com/article/10.3390/life14060677/s1, Figure S1. Rarefaction curves based on Shannon index for studied samples (Sample—tunnel_core_depth m); Table S1. Samples with estimated nitrate concentration and quantity of isolated DNA.
