# Supplementary material for: Low Bacterial Diversity and Nitrate Levels in Cores from Deep Boreholes in Pristine Karst
Source: Life (Basel). 2024 May 24;14(6):677. doi: 10.3390/life14060677 (PMC11204850; doi:10.3390/life14060677)
Supplement: Supplementary file 1 [file life-14-00677-s001.zip › life-3007530-supplementary.pdf]

Table S1. Samples with estimated nitrate concentration and quantity of isolated DNA

Sample (tunnel_core_depth m)	Main characteristics of cores (filling material / void characteristics)	Nitrate (mg/kg)	DNA (ng/µl / A_{260}/A_{280})
T1_12_89	limestone, clay / fracture	7.8±0.8	0.9 / 2.07
T1_12_101	limestone, clay / fracture	5.9±0.6	n.a.
T1_12_113	limestone, clay / fracture	12.6±0.5	n.a.
T1_12_116	limestone, clay / fracture	4.0±0.4	n.a.
T1_12_185	limestone, clay / fracture, crushed material	4.0±0.4	n.a.
T1_12_205	limestone, clay / fracture	5.9±0.6	n.a.
T1_12_207	limestone, clay / fracture	5.9±0.6	n.a.
T1_12_212	limestone, clay / fracture, crushed material	4.0±0.4	n.a.
T1_12_214	limestone, clay / fracture, crushed material	8.8±1.4	2.4 / 1.32
T1_12_215	limestone, clay / fracture, crushed material	9.7±1.0	n.a.
T1_12_221	limestone, clay / fracture, crushed material	21.1±2.2	1.9 / 1.67
T1_12_224	limestone, clay / fracture, crushed material	7.8±0.8	n.a.
T1_12_225	limestone, clay / fracture, crushed material	5.9±0.6	n.a.
T1_12_228	limestone, clay / fracture, crushed material	5.9±0.6	n.a.
T1_12_229	limestone, clay / fracture, crushed material	5.9±0.6	n.a.
T1_12_231	limestone, clay / fracture, crushed material	5.9±0.6	n.a.
T1_12_241	limestone, clay / fracture, crushed material	5.9±0.6	2.7 / 1.55
T1_13_30	clay / filled cavity	9.7±2.9	n.a.
T1_13_31	clay / filled cavity	5.9±0.7	n.a.
T1_13_32	clay / filled cavity	4.0±0.4	n.a.
T1_13_33	clay / filled cavity	4.0±0.4	n.a.
T1_13_45	clay / filled cavity	6.9±1.2	n.a.
T1_13_47	clay / filled cavity	4.0±0.4	n.a.
T1_13_48	clay / filled cavity	5.0±0.3	n.a.
T1_13_49	clay / filled cavity	5.9±0.7	n.a.
T1_13_50	clay / filled cavity	22.0±8.7	n.a.
T1_13_51	limestone, clay / fracture, crushed material	7.8±0.9	n.a.
T1_13_53	limestone, clay / fracture, crushed material	6.9±0.4	n.a.
T1_13_56	limestone, clay / fracture	5.9±0.6	n.a.
T1_13_63	limestone, clay / fracture	9.7±1.0	n.a.
T1_13_65	limestone, clay / fracture, crushed material	8.8±0.5	n.a.
T1_13_66	limestone, clay / fracture, crushed material	4.0±0.4	n.a.
T1_13_67	limestone, clay / fracture, crushed material	6.9±0.4	n.a.
T1_13_68	limestone, clay / fracture	6.9±0.4	n.a.
T1_13_69	limestone, clay / fracture, crushed material	5.9±0.6	n.a.
T1_13_113	limestone, clay / fracture	6.9±0.4	n.a.
T1_13_208	clay / filled cavity	2.2±0.2	n.a.
T1_13_214	limestone, clay / fracture	5.9±0.6	n.a.
T1_13_216	limestone, clay / fracture, crushed material	4.0±0.4	n.a.
T1_13_224	limestone, clay / fracture	2.2±0.2	n.a.
T1_13_227	limestone, clay / fracture	2.2±0.2	n.a.
T1_13_232	limestone, clay / fracture, crushed material	11.6±4.2	14.2 / 2.40
T1_13_235	clay / filled cavity	3.1±0.2	n.a.
T1_13_239	clay / filled cavity	4.0±0.4	n.a.
T1_13_244	clay / filled cavity	3.1±0.2	n.a.
T1_13_247	clay / filled cavity	3.1±0.2	n.a.
T1_13_254	clay / filled cavity	4.0±0.4	n.a.
T1_13_258	clay / filled cavity	3.1±0.2	n.a.
T1_13_261	clay / filled cavity	3.1±.0.2	n.a.
T1_13_272	clay / filled cavity	21.1±10.5	4.7 / 2.30
T1_13_277	limestone, clay / fracture, crushed material	4.0±0.4	n.a.
T1_13_283	limestone, clay / fracture	4.0±0.6	n.a.
T1_13_292	limestone, clay / fracture	5.9±0.6	1.4 / 1.61
T1_13_297	limestone, clay / fracture	5.9±0.7	n.a.
T1_13_307	limestone, clay / fracture	2.2±0.2	n.a.
T1_13_314	limestone, clay / fracture	2.2±0.2	1.8 / 1.45
T1_13_349	limestone, clay / fracture	9.7±1.0	0.7 / 1.24
T2_19_128	limestone, clay / fracture	71.2±9.2	203.7 / 2.31

T2_19_129	limestone, clay / fracture	16.3±3.0	n.a.
T2_20_7	limestone, clay / fracture	12.6±3.8	11.2 / 1.75
T2_20_15	limestone, clay / fracture	57.0±6.0	n.a.
T2_20_64	limestone, clay / fracture	11.6±2.1	n.a.
T2_20_66	limestone, clay / fracture	10.7±2.5	6.1 / 1.53
T2_20_69	limestone, clay / fracture	30.5±3.2	3.1 / 1.88
T2_20_84	limestone, clay / fracture	10.7±1.5	0.7 / 1.60
T2_20_89	limestone, flysch, clay / fracture	1503.5±157.9	n.a.
**Soil samples**	**Location**		
cabbage garden	Lokev	158.2±74.2	n.a.
garden after fertilizing	Lokev	870.1±28.1	n.a.
meadow	Šepulje	21.1±2.6	n.a.
spinach garden	Lokev	435.2±231.5	n.a.
meadow close to vineyard	Dutovlje	340.6±64.4	n.a.

n.a. – not available

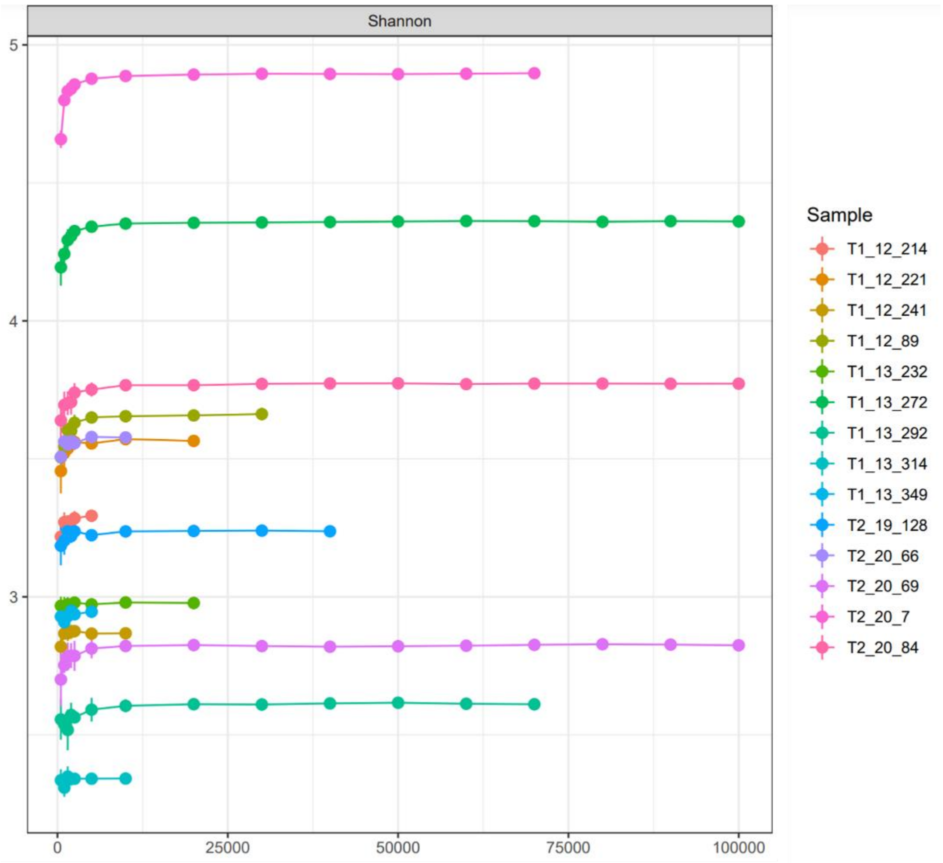

Figure S1. Rarefaction curves based on Shannon index for studied samples (Sample – tunnel_core_depth m)
